# Effects of the termination of LC_30_ imidacloprid stress on the multigeneration adaptive strategies of *Aphis glycines* population

**DOI:** 10.3389/fphys.2023.1153249

**Published:** 2023-07-31

**Authors:** Aonan Zhang, Nan Dou, Zhongcheng Qu, Yongxia Guo, WenJing Zhou, Dongxue Wu, Zhiying Lin, Min Feng, Hengjia Cui, Lanlan Han

**Affiliations:** ^1^ College of Plant Protection, Northeast Agricultural University, Harbin, Heilongjiang, China; ^2^ Qiqihar Branch of Heilongjiang Academy of Agricultural Sciences, Qiqihar City, Heilongjiang, China; ^3^ National Coarse Cereals Engineering Research Center, Key Laboratory of Low-Carbon Green Agriculture in Northeastern China, Ministry of Agriculture and Rural Affairs China and Heilongjiang Provincial Key Laboratory of Crop Pest Interaction Biology and Ecological Control, Daqing, China

**Keywords:** *Aphis glycines*, imidacloprid, recovering population, life table, transcriptome

## Abstract

*Aphis glycines* Matsumura (Hemiptera: Aphididae) is a major soybean pest that often poses a serious threat to soybean production. Imidacloprid is one of the commonly used insecticides to control the soybean aphid. To investigate the effect of termination of imidacloprid stress on the adaptive strategies of soybean aphid populations, we studied the growth, development, and related metabolism changes when the stress was terminated after 24 generations of imidacloprid stress on *A. glycines*. The results show that the *A. glycines* population accelerated its recovery and expanded its population size across generations. The longevity of the adults of the recovering population in the F12, F18, and F24 generations, respectively, was 1.11, 1.15, and 1.11 times longer than the control, while the fecundity was 10.38%, 11.74%, and 11.61% higher than that of the control. The net reproductive rate (*R*_0_) of the recovering population was always significantly higher than that of the control in the F1 to F24 generations. In addition, metabolisms related to the regulation of cell proliferation and oocyte meiosis were significantly upregulated in the recovering population. Even when the imidacloprid pressure disappeared, intergenerational stimuli still affected the adaptive strategies of soybean aphid populations. This effect was manifested as inhibiting the growth and development of the soybean aphid in the early generations and improving the fecundity of the soybean aphid in the later generations. Adaptive soybean aphid populations would surge in the absence of imidacloprid pressure. This study provides an important reference for exploring the adaptability of the *A. glycines* population under termination of stress from low lethal concentrations of imidacloprid across generations. It also provides important data for monitoring the population dynamics of *A. glycines* in the field and analyzing the degree of pharmacodynamic stress.

## 1 Introduction

The soybean aphid, *Aphis glycines* Matsumura is native to Asia and mainly distributed in the soybean growing regions of the Far East. It was discovered in North America in 2000 (Ragsdale et al., 2011; Hopper et al., 2017). It is one of the major pests that damages cultivated soybeans through piercing and sucking (Hullé et al., 2020). It has become a worldwide agricultural pest (Todd et al., 2022).

Insecticide application remains an effective management strategy for preventing outbreaks of soybean aphids (Mokbel, 2019; Aseperi et al., 2020; Valmorbida et al., 2022). The first generation of neonicotinoids imidacloprid had an excellent control effect on the soybean aphid, and rapidly dominated the insecticide market since its production (Stamm et al., 2016; Hesler and Beckendorf, 2021; Zhang et al., 2022). Although imidacloprid is effective against piercing–sucking pests, its frequent application still has negative effects. For example, *Myzus persicae* populations from different regions were reported to have shown some adaptability to imidacloprid (Devine et al., 1996). The adaptation of field aphids to neonicotinoids might be related to long-term exposure to low doses of insecticides. Plant, animal, fungal, and bacterial interactions cause concentrations of neonicotinoids to decrease gradually from the initial field application levels. Chronic exposure of aphids to neonicotinoids at lower-than-recommended concentrations, across multi-generations has been reported. Also, low doses of insecticides promote the development of resistance in heterozygotes, which can cause rapid fixation of resistance genes in the population. This results in the rapid development of resistant populations and the enhancement of population adaptability (Desneux et al., 2005; Desneux et al., 2007; Fenner et al., 2013). Also, exposures of *A. gossypii* to low doses of thiamethoxam had an intergenerational stimulative effect on the adaptability of its population. The reproduction rate of the aphid population accelerated, which peaked in a shorter time (Ullah et al., 2020a). At present, many researchers have focused on the effects of low-dose neonicotinoids stress on the growth and development of populations. Few researchers have reported on the changes in adaptive strategies of populations after the termination of low-dose insecticide stress. Will the intergenerational stimulative effect continue to affect the adaptive strategies of populations after termination of low-dose insecticide stress? Do adaptive pest populations proliferate without insecticide pressure? These are urgent questions to address for which studies are required.

The insect population life table is an effective tool in population ecology theory to describe population dynamics and analyze population adaptability (Bevill and Louda, 1999). In agricultural production, the acquisition of life table data on a species is important for the efficient management of agricultural pests (Rajotte, 2022). The age–stage life table model has the advantage of considering the relationships and differences between individuals at different developmental stages when predicting population dynamics. It also adopts a systematic identification method and relies on the model to manage population data and estimate the transfer probability between different developmental stages (Chi and Liu, 1985; Chi, 1988; Chi et al., 2020). For example, the basic data and derived parameters provided by the age–stage life table were used to predict the population adaptability of *Bactrocera cucurbitae* (Huang and Chi, 2012). Zhang et al. (2021) found that low-dose thiamethoxam reduced the adaptability of the soybean aphid population by studying the age–stage life table data. Ullah et al. (2019) also used the age–stage life table to analyze the effects of acetamiprid on the adaptive strategies of *Aphis gossypii* and found that the negative effects from the mother still inhibited the development of the offspring.

The regulation of the adaptation strategy of insect populations involves not only changes in population parameters but also changes in the corresponding metabolic regulation. Transcriptome sequencing enables the generation of a large amount of RNA expression data, screens differential genes, analyzes changes in metabolic pathways, and thus reveals the factors that influence the regulation of the adaptive strategy of insect populations (Ma et al., 2016). In this study, the effects of the termination of LC_30_ imidacloprid stress on the multigenerational adaptation strategy of the soybean aphid population were evaluated by analyzing the population parameters and development-related transcription expressions. The results serve as an important reference for understanding the changes in adaptation strategy of soybean aphid populations after the termination of imidacloprid stress. It also provides important information for monitoring the soybean aphid population dynamics in the field and analyzing the degree of pharmacodynamic stress.

## 2 Materials and methods

### 2.1 Soybean aphid populations and chemical reagents

All populations were fed dongnong bean 252 soybean plants in the laboratory with 25°C ± 1°C, a relative humidity of 65%–70%, and a photoperiod of 14:10 (L:D).

#### 2.1.1 Laboratory population

The laboratory population of *A. glycines* used in this study was originally collected from a soybean field in Xiangyang farm, Harbin, Heilongjiang, China. The population was cultured separately in the laboratory of Northeast Agricultural University since 2014 and was never exposed to any insecticides.

#### 2.1.2 Field population

The field population used in this study was also collected from a soybean field in Xiangyang in mid-July 2019. The F1–F24 generations were used in this experiment.

#### 2.1.3 Resistant population

The resistant population was derived from the laboratory population subjected to imidacloprid. The laboratory population was exposed to LC_30_ (30% lethal concentration) imidacloprid for F1–F24 generations, from which the resistant population was obtained.

#### 2.1.4 Recovering population

The recovering population used in this study was slowly recovered from the abovementioned resistant soybean aphid across generations. Specifically, exposure of the resistant population at the F24 to imidacloprid was stopped, and they were fed fresh soybean leaves for development and reproduction.

Water dispersible granules of 70% imidacloprid were purchased from North China Pharmaceutical Group Corporation, Hebei, China. Calcium nitrate, potassium nitrate, potassium dihydrogen phosphate, magnesium sulfate, disodium ethylenediaminetetraacetic acid (disodium EDTA), and streptomycin sulfate were all purchased from Shanghai Alighting Biochemical Technology Co., Ltd., Shanghai, China.

### 2.2 Feeding methods of soybean aphids in the laboratory

Dongnong bean 252 soybean plants were grown in pots (15 cm diameter × 17 cm depth), with six plants in each pot that were kept at 25°C ± 1°C, a relative humidity of 65%–70%, and a photoperiod of 14:10 h (L:D). The laboratory aphid colony was maintained in the same environmental conditions as that of the chamber which was used for plant germination. A total of 12 pots with soybean plants were placed in a large tray (L × W = 70 × 60 cm). Then, twice a week, one-third of the old aphid-infested soybean plants (i.e., the four oldest pots with an aphid infestation) were removed and replaced with new aphid-free ones. Aphids were transferred by placing infested leaves on non-infested plants. This prevented the accumulation of excessive honeydew and sooty mold and ensured the provision of a homogeneous soybean plant for the aphids to feed on (James et al., 2020).

### 2.3 Preparation of culture medium

**FIGURE 1:**
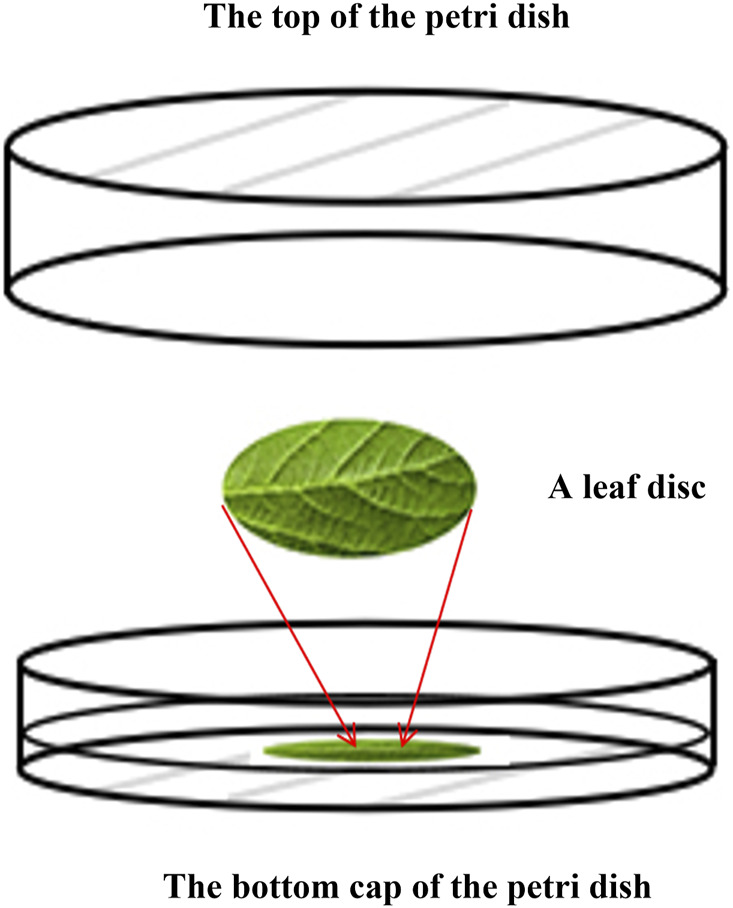
Diagram of Petri dish.

Non-toxic, transparent plastic Petri dishes (6 cm diameter × 1.5 cm height) were used to perform the bioassays involving newly hatched nymphs of *A. glycines* as well as to perform the life table study. The components of the plant nutrient concentrate used to prepare the medium were calcium nitrate (4.1 g), potassium nitrate (2.5 g), potassium dihydrogen phosphate (0.7 g), magnesium sulfate (0.6 g), 1.54% disodium EDTA aqueous solution (5.0 mL), one million units of streptomycin sulfate (0.05 g), and distilled water (5.0 L). The diluent was obtained by mixing the nutrient concentrate with distilled water at a ratio of 1:3. Agar was prepared by mixing 1% w/w agar powder with diluent and was boiled while constantly mixing. After cooling for approximately 10 min, the warm agar was poured into the Petri dishes to a depth of at least 3–4 mm. At least 10 mm distance was allowed between the top of the agar and the rim of the Petri dishes. A metal tube was used to cut leaf discs from clean, untreated leaves. The leaf discs were attached topside down on the agar medium. *A. glycines* on leaf discs fed on the bottom surface (Figure 1). Each Petri dish was then placed upside down to keep *A. glycines* in a natural feeding state.

### 2.4 Dose–response bioassay and resistance ratios

The dose–response bioassays were conducted with adults from the laboratory population, resistant population, and recovering population using the leaf dip method recommended by the Insecticide Resistance Action Committee (IRAC; http://www.irac-online.org/resources/methods.asp). Insecticidal stock solutions were prepared in 1% acetone and further diluted to different concentrations using distilled water containing 0.05% (v/v) Triton X-100, before being used in the dose–response bioassay. Fresh soybean leaf discs were immersed in solutions of a series of imidacloprid; each leaf disc was immersed in a specific concentration for 10 s and then removed from the solution to air dry. The control leaf disc was immersed in a solution of distilled water containing 0.05% (v/v) Triton X-100% and 1% acetone. The air-dried leaf discs were attached to the agar medium with the top-side facing down, and the newly hatched nymphs were placed on them. Treatment details (insecticide, concentration, and date) were recorded for each Petri dish. A small drop of distilled water was placed on the surface of the agar prior to laying the leaf on the surface to ensure that the leaf stuck to the agar surface. A total of 60 adults were used for the dose–response bioassays at each concentration; three replicates were used per concentration, and each replicate had 20 adults. Mortality was determined 24 h after exposure. The nymphs were considered dead if they were found upside down and not moving or if they did not move when prodded with a small paint brush. The toxicity of imidacloprid to adults were statistically analyzed using the concentration–mortality regression line and a log-probit model of SPSS (version 23.0, SPSS Inc., Chicago, IL, United States), and the LC_50_ and LC_30_ values were obtained. The resistance ratios of the resistant population were calculated as LC_50_ ratios between the resistant population and laboratory population. The resistance ratio of the recovering population was calculated as LC_50_ ratios between the recovering population and laboratory population. LC_50_ and LC_30_ concentrations of imidacloprid to adults of laboratory population in supplementary information (Supplementary Table S1).

### 2.5 Life table construction

The method that was followed for the construction of the life table for the laboratory population (control group) is as follows: 100 apterous adults (F0) were transferred onto 10 leaf discs using a small paint brush, with 10 apterous adults placed on each leaf disc. Each Petri dish containing a leaf disc was sealed with a close-fitting ventilated lid. After 24 h, the newly hatched nymphs (F1) produced by the selected aphid were placed in a new Petri dish with a leaf disc. Leaf discs were pre-soaked in distilled water solution containing 0.05% (v/v) Triton X-100% and 1% acetone. A newly hatched nymph was placed on each leaf disc, which served as a replicate, for a total of 100 replicates. Leaf discs were replaced every 24 h. Growth, survival, and death of individuals were observed and recorded every 24 h until all individuals died. The numbers of newly hatched aphids were recorded and then discarded to avoid repeated recording the next day. After that, the construction of the life table for the laboratory population in each generation was repeated using newly hatched aphids produced in the previous generation.

The construction of the life table for the resistant population, which followed the aforementioned method, is as follows: 100 apterous adults (F0) were transferred onto 10 leaf discs using a small paint brush, with 10 apterous adults placed on each leaf disc. A leaf disc pre-impregnated with LC_30_ imidacloprid was placed on each Petri dish. After 24 h, the surviving aphids were placed on a new leaf disc free of the chemical and pre-impregnated with distilled water solution containing 0.05% (v/v) Triton X-100% and 1% acetone; they were allowed to continue feeding to produce the next generation of adults (F1). The process of constructing the life table of the soybean aphid in each generation was similar. The concentration of LC_30_ imidacloprid used in each generation was 30% lethal for adults. The adults of each generation were fed with chemical-free leaf discs 24 h after insecticide stress.

The construction of the life table for the recovering population is as follows: the initial hatchlings of the resistant population produced by the F24 generation were recorded as the recovering F1 generation population. A single hatchling was then reared in separate Petri dishes, each of which was pre-impregnated with a distilled water solution containing 0.05% (v/v) Triton X-100% and 1% acetone for 100 replicates. Growth, survival, and death of the individuals were observed and recorded every 24 h until all individuals died. Data were recorded in the same manner as for the laboratory population.

The construction of the life table for the field population is as follows: the method of constructing the life table of the field population was similar to that of the laboratory population. They differed only in the source of the tested insects.

Preliminary results showed that there was no significant difference in the life table parameters of adjacent generations. We therefore compared and analyzed the changes in the life table parameters of F1, F6, F12, F18, and F24.

### 2.6 Life table data analysis

The age–stage–specific survival rate (*s*_*xj*_, x = age, j = stage), age-specific survival rate (*l*_*x*_), age–stage and age-specific fecundity (*m*_*x*_) were calculated as follows (Chi and Liu, 1985):

$$s_{xj}=\frac{n_{xj}}{n_{01}},$$ (1)

$$l_{x}=\sum_{j=1}^{k}s_{xj},$$ (2)

$$m_{x}=\frac{\sum_{j=1}^{k}s_{xj}f_{xj}}{\sum_{j=1}^{k}s_{xj}},$$ (3)

where *n*_01_ is the number of newly hatched nymphs, and *k* is the number of stages. The net reproductive rate (*R*_0_), intrinsic rate of increase (*r*), finite rate of increase (*λ*), and mean generation time (*T*) were calculated as follows (Goodman, 1982):

$$R_{0}=\sum_{x=0}^{\infty }l_{x}m_{x},$$ (4)

$$\sum_{x=0}^{\infty }e^{-r\left(x+1\right)}l_{x}m_{x}=1,$$ (5)

$$\lambda =e^{r},$$ (6)

$$T=\frac{1n\;R_{0}}{r}.$$ (7)

The life expectancy (*e*_*xj*_), i.e., the time that an individual of age *x* and stage *j* is expected to live, was calculated according to Chi and Su (2006) as

$$e_{xj}=\sum_{i=x}^{\infty }\sum_{y=j}^{k}s_{iy}^{\prime }.$$ (8)

where *s*_*iy*_ *’* is the probability that an individual of age *x* and stage *j* would survive to age *i* and stage *y*. Tuan et al. (2014) defined the reproductive value (*v*_*xj*_) as the contribution of individuals of age *x* and stage *j* to the future population. It was calculated as follows:

$$v_{xj}=\frac{e^{r\left(x+1\right)}}{s_{xj}}\sum_{i=x}^{\infty }e^{-r\left(i+1\right)}\sum_{y=j}^{k}s_{iy}^{\prime }f_{iy}.$$ (9)

The mean values and standard errors of the population parameters, mean longevity of the first to fourth instar nymphs and adults, adult and total pre-reproductive period, and mean fecundity were analyzed using the TWOSEX-MSChart software. The paired bootstrap test (*B* = 100,000), which was based on the percentile of differences and 95% CI of normalized distribution of differences, was used to compare the differences among treatments. The toxicity of imidacloprid to the adults was analyzed using the log-probit model of SPSS 23.0 and the concentration–mortality regression line. Graphs were generated using SigmaPlot 12.0.

### 2.7 Transcriptome sequencing

Total RNA was extracted from whole adults using TRIzol according to the method of Wu et al. (2017). The adults for transcriptome sequencing were independently obtained from the F24 generation of the laboratory, field, resistant, and recovering populations. The mRNA of poly-A tail was enriched from the total RNA of adults using Oligo dT magnetic beads. The enriched RNA was then fragmented with a fragmentation buffer, about 200 bp. The cDNA was then synthesized and purified several times using AMPure XP beads to remove small non-target segments from the reaction system. Each qualified cDNA library was sequenced and the sequencing company was Wuhan Kangce Technology Co., Ltd. The expression quantity was calculated using the SMEM algorithm of Salmon (v.0.9.1) software with the Perl script abundance_estimates_to_matrix.pl in Trinity (v.2.4.0) software. The threshold was set to | log2 (a fold changes) | > 1 and *p*-value < 0.01. KEGG enrichment analysis was performed using ClusterProfiler (v.3.8.1).

## 3 Results

### 3.1 Toxicity of imidacloprid to adults in different generations

The LC_50_ and LC_30_ values of the resistant population in F24 increased by 36.611 and 28.747 when compared with F1, respectively (Table 1). The LC_50_ and LC_30_ values of the recovering population in F24 decreased by 17.331 and 16.119 when compared with F1, respectively (Table 2).

**TABLE 1:** Toxicity of imidacloprid to adults of the resistant population in different generations.

Generation	Resistant population				
	LC_50_ (CI_95_)^a^ (mg/L)	LC_30_ (CI_95_) (mg/L)	Slope ± SE	χ^2^ (df)	*p*
F1	5.636 (0.513–9.590)	3.469 (0.115–6.985)	2.489 ± 0.865	0.444 (5)	0.994
F6	11.832 (5.025–15.773)	8.314 (2.295–12.303)	3.422 ± 0.982	0.505 (5)	0.992
F12	26.504 (24.239–28.987)	22.098 (19.600–24.168)	6.642 ± 0.946	0.481 (5)	0.993
F18	36.761 (31.486–42.354)	26.706 (20.564–31.224)	3.779 ± 0.683	0.451 (5)	0.994
F24	42.247 (36.839–47.540)	32.216 (25.358–36.928)	4.455 ± 0.832	0.39 (5)	0.996

^a^ CI_95_ is 95% confidence interval.

**TABLE 2:** Toxicity of imidacloprid to adults of the recovering population in different generations.

Generation	Recovering population				
	LC_50_ (CI_95_)^a^ (mg/L)	LC_30_ (CI_95_) (mg/L)	Slope ± SE	χ^2^ (df)	*P*
F1	41.015 (36.174–45.630)	32.095 (25.735–36.355)	4.924 ± 0.927	0.262 (5)	0.998
F6	39.564 (34.410–44.566)	30.036 (23.281–34.513)	4.382 ± 0.859	0.503 (5)	0.992
F12	33.629 (29.203–37.937)	25.5 (18.694–29.331)	4.363 ± 1.028	0.512 (5)	0.992
F18	27.644 (23.123–32.191)	19.483 (13.155–23.256)	3.451 ± 0.813	0.512 (5)	0.992
F24	23.684 (19.378–28.113)	15.976 (10.280–19.495)	3.067 ± 0.721	0.515 (5)	0.992

^a^ CI_95_ is 95% confidence interval.

### 3.2 Tolerance of soybean aphids after termination of imidacloprid stress

**FIGURE 2:**
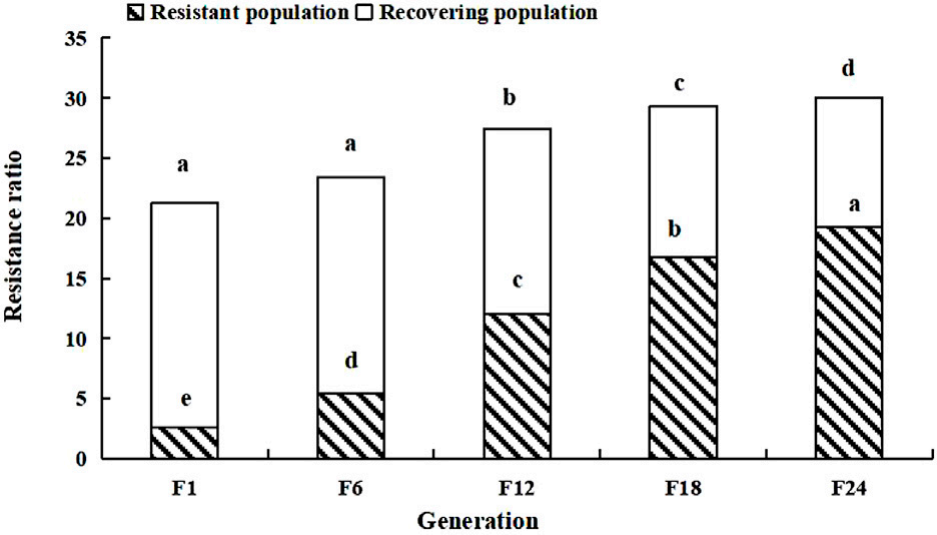
Resistance ratio of the soybean aphid population. Different lowercase letters indicate significant difference, and the significance level is 0.05.

After termination of LC_30_ imidacloprid stress, the tolerance of the soybean aphid population decreased, and the resistance ratio of the recovering population in F1 was 18.69; 0.57 lower than that in F24 of the resistant population. The resistance ratio of the recovering population in F24 decreased to 10.79; 8.47 lower than the highest resistance ratio of the resistant population (Figure 2).

### 3.3 Effects of termination of long-term imidacloprid stress on life history parameters of soybean aphid population

The termination of LC_30_ imidacloprid stress had effects on the developmental duration, lifespan of adults, and fecundity in the recovering population of the F1 generation (Table 3). The developmental time of fourth instar nymphs and the total pre-reproductive period (TPOP) of the F1 recovering population were significantly longer than that of the control, while the developmental time of the second to fourth instar nymphs and the total pre-reproductive period (TPOP) were significantly shorter than that of the resistant population. Also, the lifespan of the adults was significantly longer and the fecundity significantly higher than those of the resistant population (*p* < 0.05). The effects of termination of imidacloprid stress on the developmental duration of the soybean aphid, lifespan, and pre-reproductive period of F6 were similar to those of F1. In F12, the longest lifespan of the adults in the recovering population was 13.03 days, which was 11.37% longer than that of the control group. The fecundity of the recovering population was 1.1, 1.06, and 1.43 times higher than that of the control, field population, and resistant population, respectively. In F18, the minimum adult pre-reproductive period (APOP) of the recovering population was 0.06 days, which was 33.33%, 85.71%, and 66.67% of the control, field population, and resistant population, respectively. The fecundity of the recovering population in F24 continued to increase and was 1.12, 1.1, and 1.23 times higher than that of the control, field population, and resistant populations, respectively (Table 3).

**TABLE 3:** Effects of termination of LC_30_ imidacloprid stress on life history parameters of soybean aphid population.

Generation	Stage	Laboratory population control (mean ± SE)	Field population (mean ± SE)	Resistant population (mean ± SE)	Recovering population (mean ± SE)
F1	L1 developmental time (d)	1.22 ± 0.04 a	1.14 ± 0.03 a	1.18 ± 0.04 a	1.20 ± 0.04 a
F1	L2 developmental time (d)	1.2 ± 0.04 b	1.14 ± 0.03 b	1.44 ± 0.06 a	1.17 ± 0.04 b
F1	L3 developmental time (d)	1.13 ± 0.04 b	1.07 ± 0.03 b	1.63 ± 0.11 a	1.12 ± 0.03 b
F1	L4 developmental time (d)	1.01 ± 0.01 c	1.29 ± 0.05 b	1.53 ± 0.10 a	1.32 ± 0.05 b
F1	Mean longevity of adult (d)	11.49 ± 0.56 a	11.92 ± 0.44 a	8.22 ± 0.74 b	11.92 ± 0.44 a
F1	Adult pre-reproductive period (APOP) (d)	0.15 ± 0.04 a	0.08 ± 0.01 a	0.07 ± 0.05 a	0.08 ± 0.03 a
F1	Total pre-reproductive period (TPOP) (d)	4.62 ± 0.07 c	4.73 ± 0.01 bc	5.58 ± 0.12 a	4.86 ± 0.07 b
F1	Fecundity	43.30 ± 1.72 a	44.11 ± 1.46 a	20.14 ± 2.29 b	43.90 ± 1.41 a
F6	L1 developmental time (d)	1.22 ± 0.04 a	1.16 ± 0.04 a	1.18 ± 0.05 a	1.13 ± 0.03 a
F6	L2 developmental time (d)	1.23 ± 0.04 b	1.12 ± 0.03 c	1.48 ± 0.08 a	1.10 ± 0.03 c
F6	L3 developmental time (d)	1.10 ± 0.03 b	1.07 ± 0.03 b	1.46 ± 0.08 a	1.08 ± 0.03 b
F6	L4 developmental time (d)	1.01 ± 0.01 b	1.29 ± 0.05 a	1.34 ± 0.07 a	1.28 ± 0.05 a
F6	Mean longevity of adult (d)	11.86 ± 0.51 a	11.85 ± 0.44 a	9.89 ± 0.74 b	12.25 ± 0.42 a
F6	Adult pre-reproductive period (APOP) (d)	0.14 ± 0.04 a	0.10 ± 0.03 a	0.06 ± 0.03 b	0.04 ± 0.02 b
F6	Total pre-reproductive period (TPOP) (d)	4.66 ± 0.07 b	4.73 ± 0.06 b	5.33 ± 0.13 a	4.64 ± 0.06 b
F6	Fecundity	45.34 ± 1.93 a	46.19 ± 1.44 a	27.48 ± 2.57 b	44.14 ± 1.41 a
F12	L1 developmental time (d)	1.20 ± 0.04 ab	1.14 ± 0.03 b	1.28 ± 0.05 a	1.12 ± 0.03 b
F12	L2 developmental time (d)	1.27 ± 0.04 a	1.15 ± 0.04 b	1.29 ± 0.05 a	1.12 ± 0.03 b
F12	L3 developmental time (d)	1.09 ± 0.03 b	1.07 ± 0.03 b	1.27 ± 0.05 a	1.06 ± 0.02 b
F12	L4 developmental time (d)	1.01 ± 0.01 c	1.29 ± 0.05 a	1.16 ± 0.05 b	1.26 ± 0.04 a
F12	Mean longevity of adult (d)	11.7 ± 0.51 b	11.97 ± 0.42 ab	11.12 ± 0.49 b	13.03 ± 0.41 a
F12	Adult pre-reproductive period (APOP) (d)	0.17 ± 0.04 a	0.08 ± 0.03 b	0.04 ± 0.02 bc	0.02 ± 0.01 c
F12	Total pre-reproductive period (TPOP) (d)	4.70 ± 0.07 b	4.73 ± 0.06 b	5.03 ± 0.08 a	4.59 ± 0.06 b
F12	Fecundity	42.95 ± 1.71 b	44.61 ± 1.47 ab	33.19 ± 1.32 c	47.41 ± 1.44 a
F18	L1 developmental time (d)	1.18 ± 0.04 a	1.14 ± 0.03 ab	1.23 ± 0.05 a	1.11 ± 0.03 b
F18	L2 developmental time (d)	1.29 ± 0.05 a	1.14 ± 0.04 b	1.20 ± 0.05 ab	1.13 ± 0.03 b
F18	L3 developmental time (d)	1.10 ± 0.03 a	1.09 ± 0.03 a	1.16 ± 0.05 a	1.07 ± 0.03 a
F18	L4 developmental time (d)	1.01 ± 0.01 c	1.29 ± 0.05 b	1.44 ± 0.06 a	1.26 ± 0.04 b
F18	Mean longevity of adult (d)	11.57 ± 0.50 b	11.88 ± 0.39 ab	11.40 ± 0.36 b	13.27 ± 0.40 a
F18	Adult pre-reproductive period (APOP) (d)	0.18 ± 0.04 a	0.07 ± 0.03 b	0.09 ± 0.04 b	0.06 ± 0.02 b
F18	Total pre-reproductive period (TPOP) (d)	4.73 ± 0.07 b	4.73 ± 0.06 b	5.15 ± 0.09 a	4.64 ± 0.06 b
F18	Fecundity	42.68 ± 1.64 b	44.17 ± 1.39 a	35.61 ± 1.44 c	47.69 ± 1.40 a
F24	L1 developmental time (d)	1.18 ± 0.04 ab	1.12 ± 0.03 b	1.28 ± 0.05 a	1.14 ± 0.04 b
F24	L2 developmental time (d)	1.28 ± 0.04 a	1.17 ± 0.04 ab	1.22 ± 0.05 ab	1.16 ± 0.04 b
F24	L3 developmental time (d)	1.11 ± 0.03 a	1.09 ± 0.03 a	1.19 ± 0.05 a	1.09 ± 0.03 a
F24	L4 developmental time (d)	1.01 ± 0.01 b	1.27 ± 0.05 a	1.36 ± 0.06 a	1.26 ± 0.04 a
F24	Mean longevity of adult (d)	12.19 ± 0.49 b	11.94 ± 0.37 b	11.38 ± 0.40 b	13.48 ± 0.40 a
F24	Adult pre-reproductive period (APOP) (d)	0.15 ± 0.04 ab	0.07 ± 0.03 b	0.18 ± 0.04 a	0.11 ± 0.03 ab
F24	Total pre-reproductive period (TPOP) (d)	4.72 ± 0.07 b	4.73 ± 0.05 b	5.23 ± 0.08 a	4.77 ± 0.07 b
F24	Fecundity	43.31 ± 1.71 bc	44.08 ± 1.36 b	39.45 ± 1.38 c	48.34 ± 1.33 a

Standard errors were estimated using 100,000 bootstrap resampling. A paired bootstrap test at the 5% significance level was used to detect differences between treatments. *Note*: L1 is the first instar nymph, L2 is the second instar nymph, L3 is the third instar nymph, and L4 is the fourth instar nymph.

### 3.4 Effects of termination of long-term imidacloprid stress on reproductive parameters of soybean aphid population

The termination of LC_30_ imidacloprid stress affected related reproductive parameters of the recovering population in F1 (Table 4). The intrinsic rate of increase (*r*) and finite rate of increase (*λ*) of the recovering population were significantly higher than those of the resistant population. The net reproductive rate (*R*_0_) of the recovering population was 1.13 and 3.61 times higher than that of the control and resistant populations, respectively. After termination of imidacloprid stress, the mean generation time (*T*) of the recovering population was significantly shorter than that of the resistant population, but significantly longer than that of the control. The intrinsic rate of increase (*r*), the finite rate of increase (*λ*), and net reproduction rate (R_0_) of the recovering population increased by 2.50%, 1.16%, and 1.61%, respectively, when compared with the corresponding parameter values of the F1. The intrinsic rate of increase (*r*), the finite rate of increase (*λ*), and net reproduction rate (R_0_) of the recovering population in F12 increased by 2.22%, 0.96%, and 9.62% when compared with the F6 generation, respectively. The net reproductive rate (R_0_) of the recovering population in F18 was the highest, which was 1.26, 1.11, and 1.89 times higher than that of the control, field, and resistant populations, respectively. The net reproductive rate (R_0_) of the recovering population in F24 was significantly higher than that of the control group and field population (Table 4).

**TABLE 4:** Effects of termination of LC_30_ imidacloprid stress on the reproductive parameters of soybean aphid population.

Generation	Parameters	Laboratory population control (mean ± SE)	Field population (mean ± SE)	Resistant population (mean ± SE)	Recovering population (mean ± SE)
F1	*r*	0.438 ± 0.008 a	0.445 ± 0.006 a	0.239 ± 0.015 b	0.440 ± 0.006 a
F1	*λ*	1.549 ± 0.012 a	1.560 ± 0.009 a	1.271 ± 0.019 b	1.552 ± 0.009 a
F1	*R*_0_	37.24 ± 2.109 b	41.9 ± 1.686 a	11.68 ± 1.651 c	42.14 ± 1.600 a
F1	*T*	8.26 ± 0.080 c	8.40 ± 0.083 bc	10.26 ± 0.215 a	8.51 ± 0.095 b
F6	*r*	0.425 ± 0.007 a	0.452 ± 0.005 a	0.288 ± 0.014 b	0.451 ± 0.005 a
F6	*λ*	1.529 ± 0.011 a	1.571 ± 0.008 a	1.333 ± 0.019 b	1.570 ± 0.008 a
F6	*R*_0_	39.45 ± 2.267 b	44.34 ± 1.649 a	17.86 ± 2.111 c	42.82 ± 1.559 a
F6	*T*	8.66 ± 0.099 b	8.40 ± 0.084 cd	10.02 ± 0.213 a	8.33 ± 0.079 d
F12	*r*	0.429 ± 0.007 a	0.443 ± 0.006 a	0.366 ± 0.010 b	0.461 ± 0.005 a
F12	*λ*	1.536 ± 0.011 a	1.557 ± 0.009 a	1.441 ± 0.014 b	1.585 ± 0.007 a
F12	*R*_0_	37.80 ± 2.050 b	42.83 ± 1.664 a	24.89 ± 1.744 c	46.94 ± 1.505 a
F12	*T*	8.47 ± 0.096 b	8.49 ± 0.084 b	8.79 ± 0.109 a	8.36 ± 0.076 b
F18	*r*	0.430 ± 0.007 a	0.438 ± 0.005 a	0.368 ± 0.011 b	0.457 ± 0.005 a
F18	*λ*	1.537 ± 0.011 a	1.550 ± 0.008 a	1.445 ± 0.015 b	1.579 ± 0.008 a
F18	*R*_0_	37.56 ± 2.000 c	42.40 ± 1.592 b	24.93 ± 1.919 d	47.21 ± 1.463 a
F18	*T*	8.43 ± 0.103 b	8.55 ± 0.082 ab	8.74 ± 0.126 a	8.44 ± 0.080 b
F24	*r*	0.426 ± 0.007 a	0.439 ± 0.005 a	0.373 ± 0.009 b	0.449 ± 0.005 a
F24	*λ*	1.531 ± 0.011 a	1.551 ± 0.008 a	1.453 ± 0.013 b	1.567 ± 0.008 a
F24	*R*_0_	38.11 ± 2.058 b	42.32 ± 1.564 b	29.19 ± 2.008 c	47.37 ± 1.470 a
F24	*T*	8.54 ± 0.099 b	8.54 ± 0.075 b	9.04 ± 0.103 a	8.59 ± 0.009 b

Standard errors were estimated using 100,000 bootstrap resampling. A paired bootstrap test at the 5% significance level was used to detect differences between treatments. *Note*: *r* is intrinsic rate of increase, *λ* is finite rate of increase, *R*_0_ is net reproductive rate, and *T* is mean generation time.

### 3.5 Effects of termination of long-term imidacloprid stress on age–stage survival rate (*s*_*xj*_) and fertility of soybean aphid population

**FIGURE 3:**
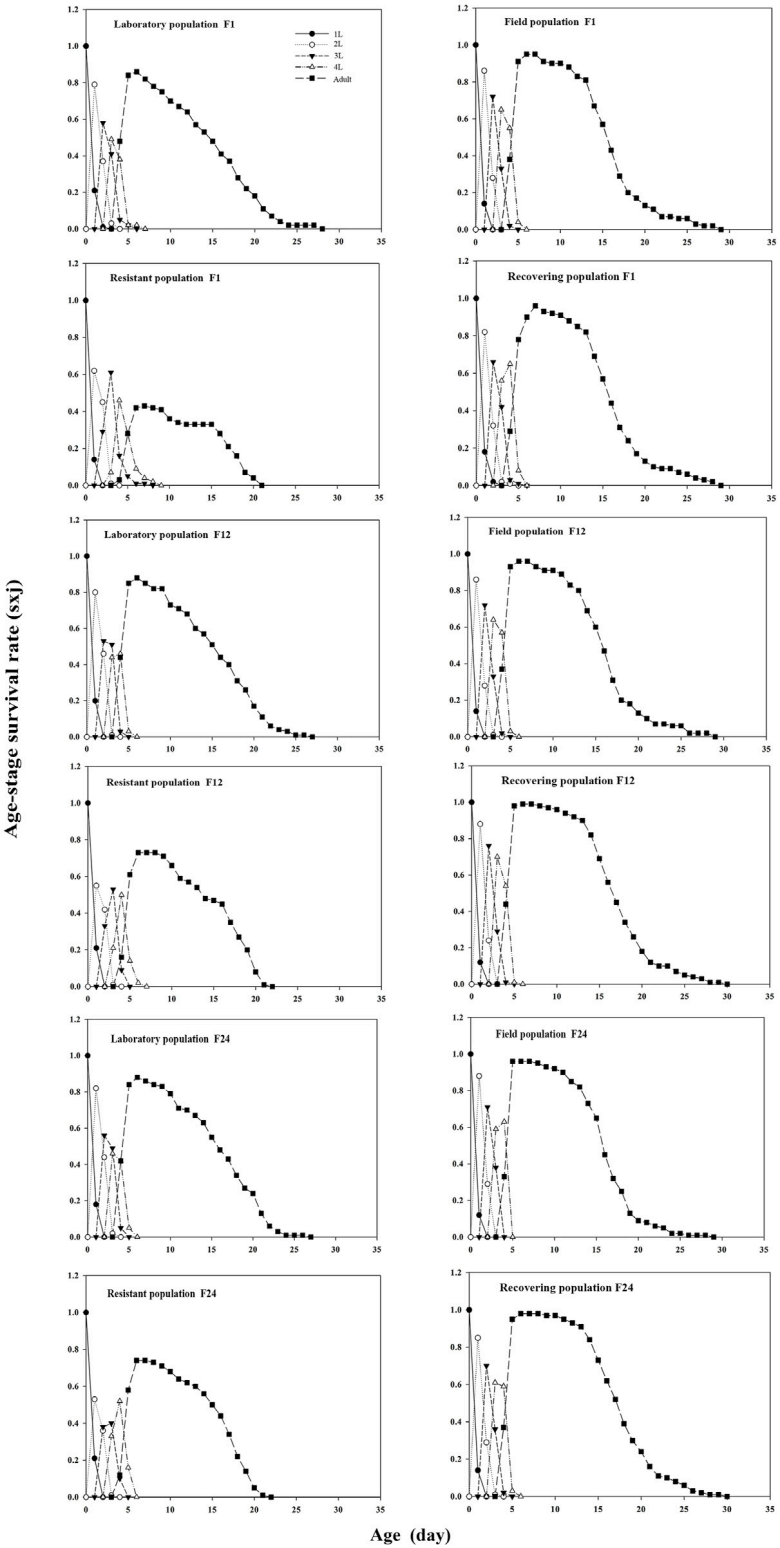
Effects of termination of LC_30_ imidacloprid stress on the age–stage survival rate (*s*_*xj*_) of the soybean aphid population. *Note*: 1L is the first instar nymphs, 2L is the second instar nymphs, 3L is the third instar nymphs, and 4L is the fourth instar nymphs.

Results showed that the age–stage survival rate (*s*_*xj*_) of the aphids overlapped (Figure 3). The probability of newly hatched nymphs reaching the adult stage for the recovering population in F1 was 0.96, while that for the control, field, and resistant populations was 0.86, 0.95, and 0.58, respectively. In F1, the peak of adults in the recovering population appeared on the seventh day, 1 day later than that of the control. The age–stage survival rate (*s*_*xj*_) of the recovering population in F12 was higher than that in F1. The probability of the soybean aphid of the recovering population successfully entering the adult stage was 0.99, which was 0.03 higher than that in the F1. In F24, the probability of the recovering population successfully entering the adult stage was 0.98, 0.1, 0.02, and 0.24 higher than that of the control, field, and resistant populations, respectively.

**FIGURE 4:**
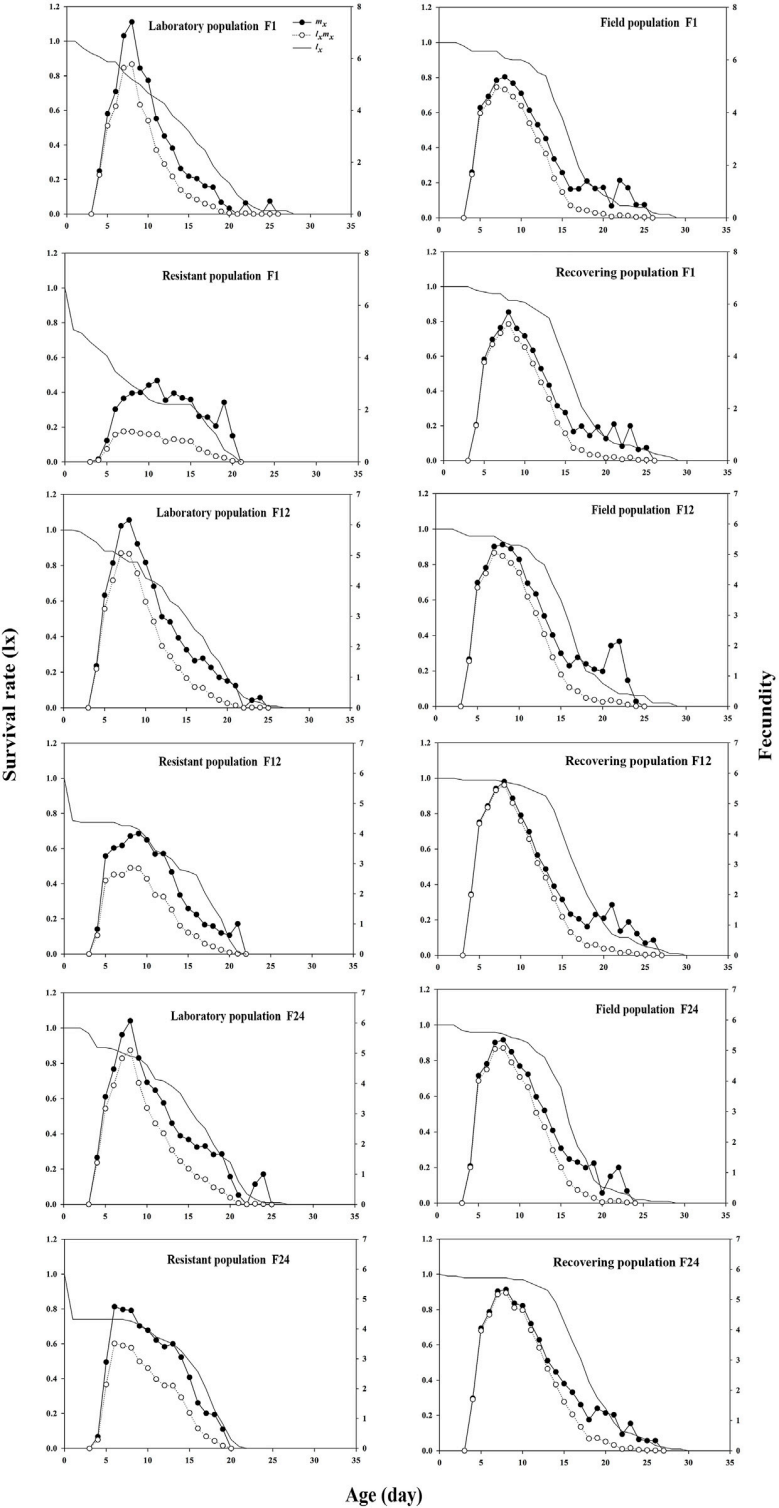
Effects of termination of LC_30_ imidacloprid stress on the age specific survival rate (*l*_*x*_) and fecundity (*m*_*x*_) of soybean aphid.

The survival curves of the control, field, and recovering populations showed a convex trend. However, the daily survival rate curve of the resistant population decreased sharply within 24 h, with the curve following a concave trend (Figure 4). The age-specific fecundity peak (*m*_*x*_) of the recovering population in F1 was 5.69, 2.57 and 0.33 higher than that of the resistant and field populations, respectively, but 1.72 lower than that of the control. The *m*_*x*_ peak of the recovering population in F24 was 5.32, which was 0.58 higher than that of the resistant population, but 0.75 and 0.02 lower than that of the control and field population, respectively (Figure 4). In addition, termination of LC_30_ imidacloprid stress significantly improved the reproductive value (*v*_*xj*_) (Supplementary Figure S1) and life expectancy (*e*_*xj*_) (Supplementary Figure S2) of the recovering population.

### 3.6 Differentially expressed genes

**FIGURE 5:**
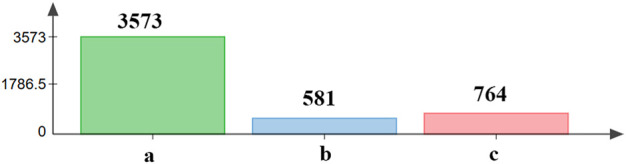
Analysis of differentially expressed genes between the recovering population and other populations. a—differentially expressed genes between the recovering population and laboratory population. b—differentially expressed genes between the recovering population and resistant population. c—differentially expressed genes between the recovering population and field population.

We found that the number of differentially expressed genes between the recovering population and the laboratory population was the largest, which was 3,573. There were 581 different genes between the recovering population and resistant population, and 764 different genes between the recovering population and field population (Figure 5).

### 3.7 KEGG pathway enrichment analysis of differentially expressed genes

**FIGURE 6:**
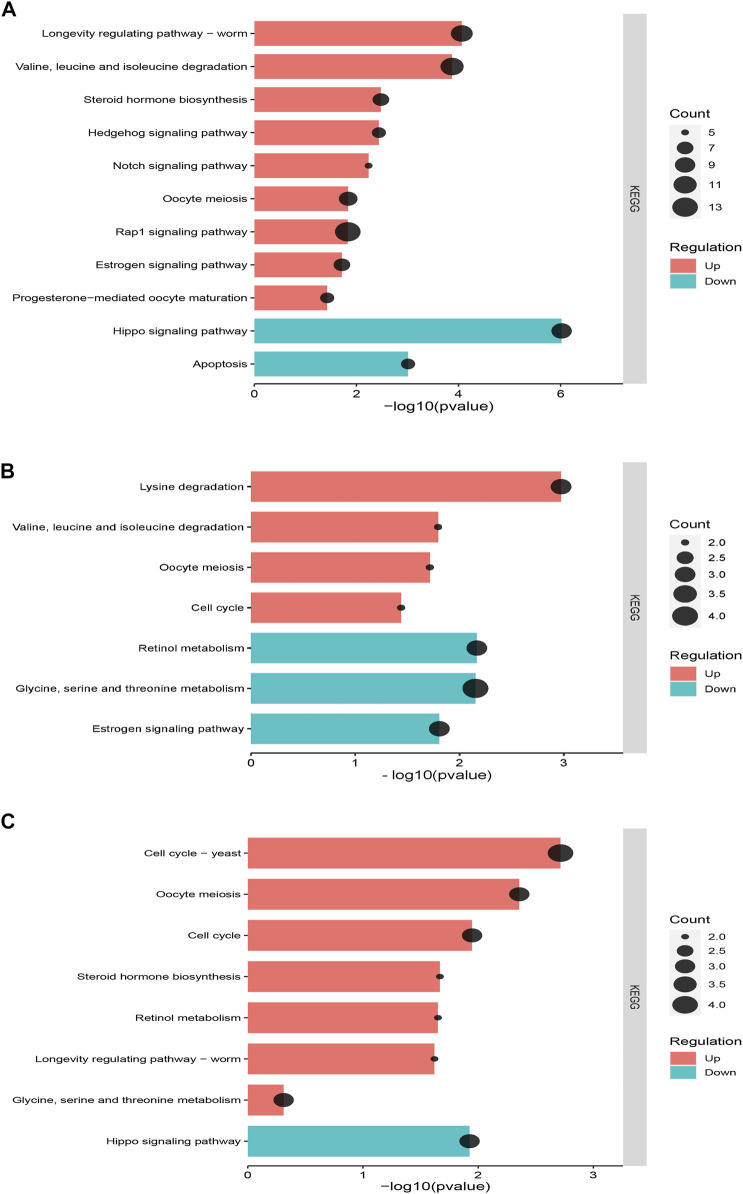
KEGG pathway enrichment analysis of differentially expressed genes between the recovering population and other populations. **(A)** KEGG pathway enrichment analysis between the recovering population and laboratory population. **(B)** KEGG pathway enrichment analysis between the recovering population and field population. **(C)** KEGG pathway enrichment analysis between the recovering population and resistant population. The horizontal axis represents the enrichment significance, which is represented by −log10 (*p*-value). The vertical axis represents the enriched KEGG pathway. The dot size indicates the number of differential genes contained in the KEGG pathway.

The enrichment analysis of KEGG pathways between the recovering population and laboratory population showed that all differentially expressed genes were distributed in 119 metabolic pathways. The significantly upregulated pathways related to population development and reproduction were longevity-regulating pathway—worm (ko04015), oocyte meiosis (ko04114), valine, leucine, and isoleucine degradation (ko00280), steroid hormone biosynthesis (ko00140), progesterone-mediated oocyte maturation (ko04914), and Rap1 signaling pathway (ko04015), Hedgehog signaling pathway (ko04340), and Notch signaling pathway (ko04330), all involved in the regulation of cell proliferation. The downregulated pathway was the Hippo signaling pathway (ko04391), which could inhibit cell growth and apoptosis (ko04214) (Figure 6A). All differentially expressed genes between the recovering population and field population were distributed in 40 metabolic pathways. The significantly upregulated pathways related to population development and reproduction were lysine degradation (ko00310), valine, leucine, and isoleucine degradation (ko00280), oocyte meiosis (ko04114), and cell cycle (ko04110). The downregulated pathways were glycine, serine, and threonine metabolism (ko00260), estrogen signaling pathway (ko04915), and retinol metabolism (ko00830) (Figure 6B). All differentially expressed genes between the recovering and resistant populations were distributed in 41 metabolic pathways, among which significantly upregulated pathways related to population development and reproduction were oocyte meiosis (ko04114), cell cycle (ko04110), glycine, serine, and threonine metabolism, and longevity regulating pathway—worm (ko04015). The downregulated pathway was the Hippo signaling pathway (ko04391) that inhibited cell growth (Figure 6C).

## 4 Discussion

Multi-generations of pests frequently exposed to insecticides could lead to a decrease in sensitivity (Pathan et al., 2008; Mansoor et al., 2017). In this study, results showed that the tolerance of the soybean aphid to imidacloprid of resistant populations increased over generations, while that of recovering populations decreased after termination of LC_30_ imidacloprid stress. A study found that the ability of parents to cope with stress was limited, and once the stress intensity exceeded the limit, the negative effects of the stress were passed on to offspring. These negative effects were reflected in the effects on the developmental time of the individual, fertility, and longevity of the offspring (Cao et al., 2021). We found that the termination of LC_30_ imidacloprid stress prolonged the developmental time and the total pre-reproductive period (TPOP) of the recovering population in F1 when compared to that of the control. It indicated that even in the absence of imidacloprid stress, the negative effects of the stress on soybean aphid parents were still transmitted to the offspring, which weakened the biological fitness of the population. A similar finding on the effects of acetamiprid stress on the biological fitness of the *Aphis gossypii* was reported (Ullah et al., 2020b). Our results showed that the longevity of adults and fecundity of the recovering population exceeded those of the laboratory population, from the F12. These results showed that the soybean aphid population accelerated its recovery after removal of imidacloprid stress, indicated by an increase in the population size across generations. This might be because the soybean aphid parents no longer required to use more energy to improve its tolerance to stress, and the offspring could obtain more resources for development and reproduction (Rugno et al., 2016; Tang et al., 2019; Cao et al., 2021).

The physiological and behavioral changes in the recovering population occurred after the removal of low-dose imidacloprid stress. The soybean aphids shortened the time required to enter the reproductive stage and quickly produced offspring, which promoted their recovery and adaptability. In addition, the life history parameters of the recovering population were similar to those for the field population, which might be related to the long-term exposure of the field population to low doses of imidacloprid.

Insecticides are degraded by bacteria, fungi, microorganisms, animals, and the natural environment in the field after application, which results in the lowering of the initial concentrations applied (Desneux et al., 2005; Desneux et al., 2007). Exposure of the soybean aphids to low doses of insecticides in the field was aided by the drifting and degradation of insecticides (Rix et al., 2016). Our results showed that low-dose imidacloprid had a stimulating effect on soybean aphids from the F12 generation, as the reproductive capacity of the recovering population was significantly higher than that of the laboratory population. This faster increase of the population may hinder the control of the soybean aphid in the field, seriously threaten the production of soybean and other crops, and might lead to an early outbreak of the soybean aphids in the field. Therefore, it is necessary to monitor the dynamics of soybean aphid populations and determine the type and dosage of insecticides to be applied, according to their developmental and reproductive characteristics (Rugno et al., 2015; Gordy et al., 2021; Holland et al., 2021).

We found out that the mean generation time (*T*) of the recovering population in F1 was still longer than that of the control even after the removal of imidacloprid stress, indicating that the low dose of imidacloprid inhibited the development of the soybean aphid population, but the inhibitory effect gradually weakened over generations. The ability of the recovering population to reproduce increased dramatically, and as generations passed, it diverged further from the laboratory population. This was probably related to the intergenerational effect of low-dose imidacloprid on the soybean aphid population (Peng et al., 2019). The soybean aphid recovered its adaptability across generations after the removal of imidacloprid stress. The time required for soybean aphids to enter the adult stage gradually advanced in each generation, and the proportion of soybean aphid individuals increased across generations.

The results also showed that the probability of a newly born nymph reaching the adult stage of the recovering population was the highest in F1. This indicated that removal of the low-dose imidacloprid stress accelerated the adaptive recovery of the soybean aphid population. In F24, the reproductive value in the recovering population exceeded that of the field and resistant populations. The fertility of the soybean aphid population increased slowly across generations after removal of imidacloprid stress. The reproductive value of the recovering population fluctuated in the late life history of the soybean aphid, which was probably related to their longevity. In addition, the reproductive value (*v*_*xj*_) and life expectancy (*e*_*xj*_) of the recovering population were very similar to those of the field soybean aphid population, which may be due to the effect of pharmacodynamic stress on the field population. When imidacloprid stress was terminated, the adaptive strategies of the soybean aphid population were adjusted across generations. The growth and development of the soybean aphid in the earlier generation were still inhibited, but the fecundity and population growth rate increased greatly in later generations. The stimulation of early generations of insects plays an important role in the induction of individual phenotypes and even intergenerational types in offspring. These phenotypes are mainly related to development, longevity, and fecundity (Xing et al., 2019; Zhao et al., 2019). Insects with frequent generations and relatively short life cycle, such as soybean aphids, are easily stimulated during their development. Meanwhile, maternal stress not only affects contemporary individuals but also affects the differential expression of developmental pathways in offspring through intergenerational effects (Zhao et al., 2017; Tran et al., 2018; Peng et al., 2019).

Compared with the other populations after termination of imidacloprid stress, a large number of upregulated pathways in the recovering population were mainly related to cell division, cell proliferation, oocyte meiosis and maturation, and amino acid metabolism. Cell division and proliferation are fundamental to the growth and reproduction of organisms (Belluccini et al., 2021). The Hedgehog signaling pathway, Notch signaling pathway, and Rap1 signaling pathway co-regulate cell fate, proliferation, and differentiation in the recovering population (Wang et al., 2010). The activation of these signaling pathways might promote the rapid proliferation of oocytes and the development and maturation of embryos, which might be the reason why the fertility of the recovering population was higher than that of the other populations. In the absence of imidacloprid stress, the activated amino acid metabolism in the recovering population is indicated by the significant upregulation of the metabolic pathways of valine, leucine, and isoleucine. These play important roles in the energy supply for population reproduction and the synthesis of large amounts of growth hormones (Hurley and Bryson, 2022), which ensures that the soybean aphid has sufficient energy to resist the negative effects from the mother. The nutrients and energy that offspring received from their mothers were also used more for development and reproduction, which might be one of the reasons for the increased fertility of the recovering population. In addition, the longevity of adults of the recovering populations was significantly higher than that of the other populations, which might be related to the upregulated expression of the longevity regulation pathway in the recovering population.

Our study serves as an important reference for understanding the changes in adaptation strategies of soybean aphids across generations when their exposure to low-dose imidacloprid stress is terminated. It also provides important data for monitoring the population dynamics of the soybean aphid in the field and analyzing the degree of pharmacodynamic stress.

## 5 Conclusion

This study investigated the effects of termination of imidacloprid stress on the development, reproduction, and metabolism of the soybean aphid. We found that despite the absence of imidacloprid pressure, intergenerational stimuli still affected the adaptive strategies of the recovering population. This effect was manifested as inhibiting the growth and development of individuals in the early generations and improving the fecundity in the later generations. Adaptive soybean aphid populations would surge in the absence of imidacloprid pressure. This study provides an important reference for exploring the adaptability of the *A. glycines* population under termination of stress from low lethal concentrations of imidacloprid, across generations.

## Data Availability

The raw data supporting the conclusion of this article will be made available by the authors, without undue reservation.
