# Supplementary figures and images for: Effects of the termination of LC30 imidacloprid stress on the multigeneration adaptive strategies of Aphis glycines population
Source: Front Physiol. 2023 Jul 31;14:1153249. doi: 10.3389/fphys.2023.1153249 (PMC10424448; doi:10.3389/fphys.2023.1153249)

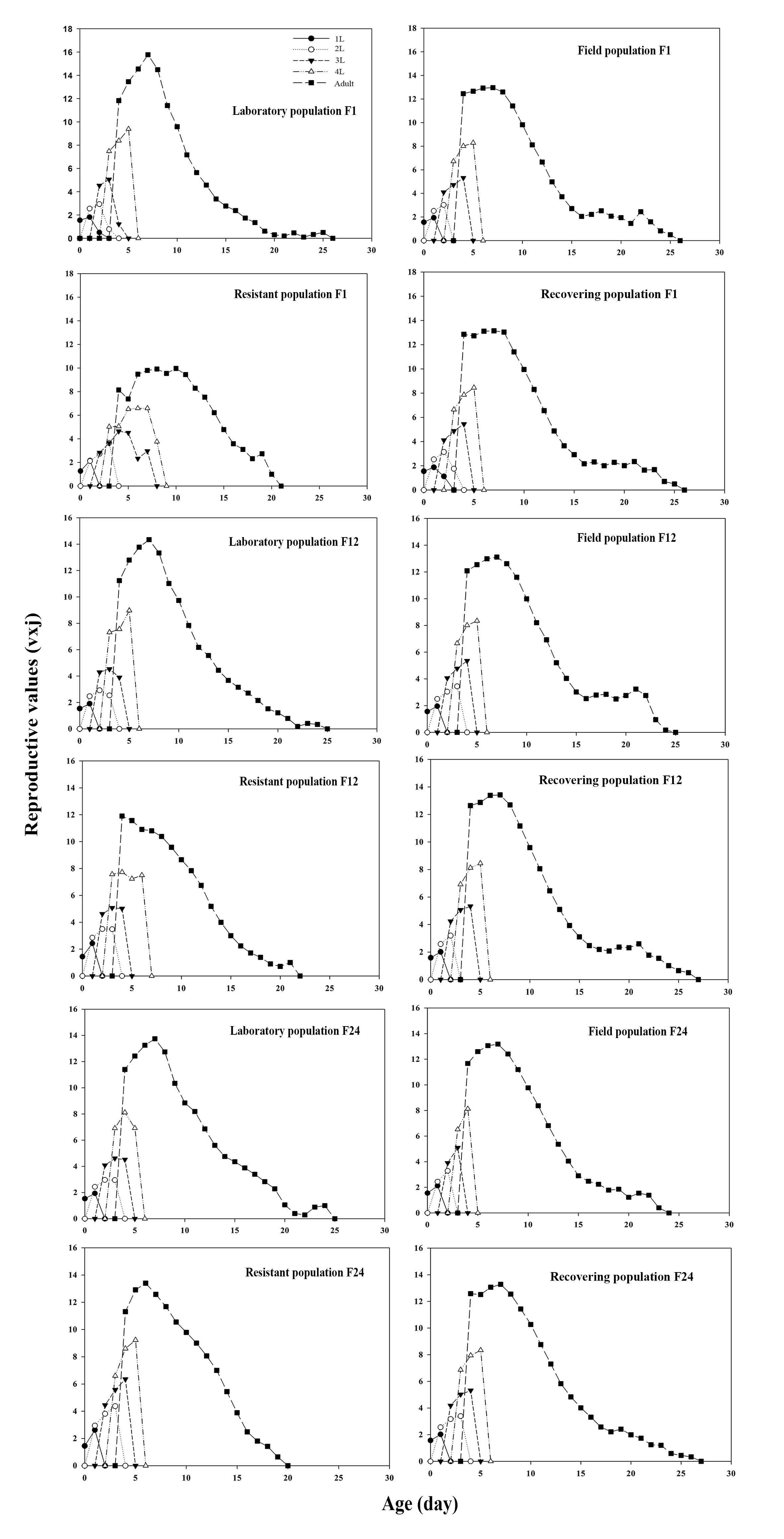

Supplement: Supplementary file 2 [file Image1.JPEG]

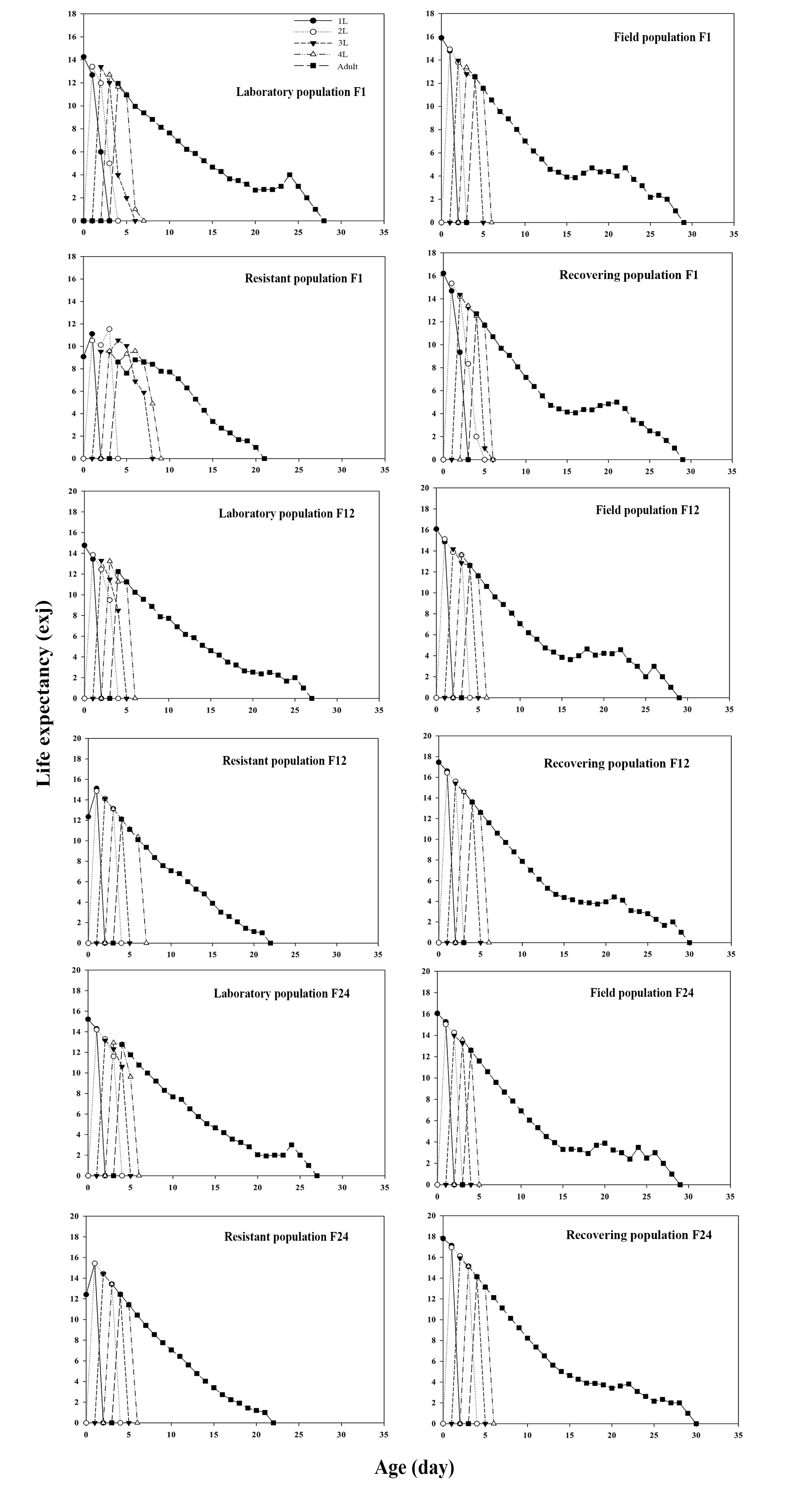

Supplement: Supplementary file 3 [file Image2.JPEG]
